# Response Rate and Impact on Lipid Profiles of Obeticholic Acid Treatment for Patients with Primary Biliary Cholangitis: A Meta-Analysis

**DOI:** 10.1155/2021/8829510

**Published:** 2021-01-15

**Authors:** Yuan Gao, Li Li, Bei Li, Yutao Zhan

**Affiliations:** ^1^Department of Rheumatism Medicine, Beijing Tongren Hospital, Capital Medical University, Beijing 100730, China; ^2^Department of Gastroenterology, Beijing Tongren Hospital, Capital Medical University, Beijing 100730, China

## Abstract

**Background:**

Up to 40% of patients with primary biliary cholangitis (PBC) have an inadequate response to ursodeoxycholic acid (UDCA). Obeticholic acid (OCA) is considered the addition of treatment, but the response rate based on commonly referenced biochemical response criteria and lipids' impact was unclear. Previous studies reported inconsistency results partially due to small sample size. Therefore, we performed a meta-analysis and aimed to explore OCA treatment's response rate and effect on lipids' profiles in PBC patients.

**Methods:**

We performed PubMed, Embase, and Cochrane controlled trials register (updated to JUN 2019) databases and manual bibliographical searches for randomized controlled trials reporting on OCA treatment in PBC patients. Two researchers independently extracted data and assessed the risk of bias of studies. We calculated risk ratio (RR) for the overall complete response rate, and the standardized mean difference (SMD) for the serum lipids changes after OCA treatment, all with 95% confidence intervals (CIs) using fixed-effects models. We registered this meta-analysis with PROSPERO (registration number: CRD42020148550).

**Results:**

Three trials, with 265 patients, were selected for the analysis. OCA was superior to placebo in PBC patients (RR, 1.48; 95% CI, 1.15–1.90). OCA's pooled treatment response rate was 65% (95% CI, 56%–74%), corresponding to Paris I criteria. Besides, OCA significantly decreased total cholesterol (*P*=0.02) with no heterogeneity (*P*=0.87, *I*^2^ = 0%) and high-density lipoprotein levels (*P* < 0.05) with no heterogeneity (*P*=0.82, *I*^2^ = 0%).

**Conclusions:**

This meta-analysis demonstrated that OCA was a promising additional treatment for PBC patients and might reduce serum cholesterol levels. The longer follow-up studies are needed to give more evidence.

## 1. Introduction

Primary biliary cholangitis (PBC, also known as primary biliary cirrhosis) is a chronic progressive autoimmune liver disease that causes ductopenia, cholestasis, and fibrosis [1–3]. Eventually, it leads to end-stage liver disease and death. The prevalence rates range from 1.91 to 118.75 cases per 100,000 globally and increase yearly tendency [4, 5]. Appropriate treatment could improve the prognosis of patients with PBC [6]. Ursodeoxycholic acid (UDCA) is a first-line treatment for PBC [7]. It can improve liver biochemistries [8], histological progression [9], and delay the time to liver transplantation [10]. Nevertheless, about up to 40% of PBC patients had an inadequate UDCA response [9, 11]. Therefore, there is an urgent need for developing new treatment options for patients with PBC.

Obeticholic acid (OCA) is a novel bile acid analog [12]. Several studies suggested that OCA could improve liver biochemical and immunologic markers for PBC [13–15]. OCA treatment response rate based on the primary endpoint was 46%-47% in the study reported by Nevens et al. [16]. However, the primary endpoint was defined as the alkaline phosphatase (ALP) level less than 1.67 times the upper limit of the normal range, with a reduction of at least 15% from baseline and a normal total bilirubin level [14]. It was not commonly referenced biochemical treatment response criteria recommended in the guidelines [7, 12]. OCA treatment's response rate based on commonly referenced biochemical treatment response criteria [13–15] is essential for physicians because the common criteria as a standardized ruler could help compare the efficacy between treatments indirectly and make the best treatment options in clinical practice.

Moreover, patients with PBC have abnormal lipids metabolism [16], and OCA could impact lipids profile. The results are inconsistent in previous studies, partially due to the small sample size [13–15]. The patients with PBC are rare [2], but meta-analyses could overcome the shortcoming by pooling studies. Therefore, we did a meta-analysis and aimed to examine the response rate based on commonly referenced biochemical response criteria and the impact on OCA treatment's lipids' metabolism in patients with PBC.

## 2. Methods

### 2.1. Search Strategy and Selection Criteria

We registered this meta-analysis with PROSPERO (registration number: CRD42020148550).

We searched electronic databases, including PubMed, Embase, and Cochrane controlled trials register (updated to JUN 2019), for “primary biliary cholangitis, primary biliary cirrhosis, PBC” in combination with the following terms: “obeticholic acid, OCA, FXR, FGF-19, FGF-15, INT-747, 6-ECDCA.” Furthermore, we manually searched all review articles, conference literature, and articles in the reference lists. We presented an electronic search strategy for the PubMed database (Supplementary 1).

The inclusion criteria included PBC, defined as the patients met at least 2 of the following three diagnostic factors: increased ALP levels for at least six months, positive AMA titer (>1 : 40 titer on immunofluorescence or M2 positive by ELISA) or PBC-specific antinuclear antibodies (antinuclear dot and nuclear Rim positive), and liver biopsy consistent with PBC [1], randomized controlled trials, sufficient data on the outcomes, or the data used to perform their calculations. There was no language restriction. We excluded overlap PBC/autoimmune hepatitis syndrome and duplicate reports from the analysis.

### 2.2. Outcomes Measured

The overall complete response rate was defined as alkaline phosphatase (ALP) <3x upper limit of the normal range (ULN) and aspartate transaminase (AST) <2x ULN and bilirubin <1 mg/dL (Paris I criteria) [13]. The changes in serum lipids in PBC patients before and after OCA treatment, total cholesterol, high-density lipoprotein cholesterol (HDL), low-density lipoprotein cholesterol (LDL), and triglycerides, were also included.

### 2.3. Data Extraction

Two authors (Yuan Gao and Bei Li) independently searched the literature and identified studies for the review. We resolved any disagreement by consensus. We extracted the following data from every included study: name of the first author, published year, the number of patients, mean age, female percentage, duration of treatment, referenced biochemical response criteria, and lipids level (total cholesterol, HDL, LDL, and triglycerides).

### 2.4. Data Analysis

We used RevMan 5.3 software (The Nordic Cochrane Center, The Cochrane Collaboration) and the “meta” and “metafor” packages of RStudio software (Version 3.5.1). We used the standardized mean difference (SMD) for the serum lipids changes after OCA treatment because the normal reference ranges were different in the included studies and the risk ratio (RR) for the overall complete response rate, all with 95% confidence intervals (CIs). We pooled the overall complete response rate. We assessed statistical heterogeneity between data using the *I*^2^ statistic. We used a fixed-effects model because the heterogeneity test showed *I*^2^ < 50% and *P* > 0.10. The study by Kowdley et al. [14] reported medians (Q1 and Q3) values of measurements, so we use a formula to recalculate means and variances [15]. Nevens et al. [16] used curve plots to show the changes in lipid values with means and standard deviations; we got the plot's value by cross-matching the measurement axis. We used a leave-one-out method to evaluate sensitivity analysis.

### 2.5. The Risk of Bias Evaluation of the Included Studies

Two researchers (Yuan Gao and Li Li) assessed the risk of bias of the included studies independently based on Cochrane risk of bias criteria for RCT. Each quality item was graded as low risk, high risk, or unclear risk.

Because we had less than ten trials in the meta-analysis, we did not perform a funnel plot.

## 3. Results

### 3.1. Description of the Selected Studies

**Figure 1:**
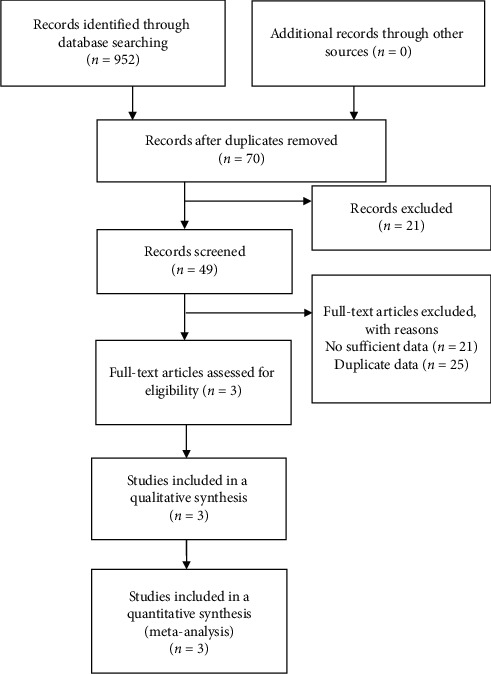
Flow chart of trial selection.

The search strategy generated 952 articles. Three articles were selected for the analysis (Figure 1). Hirschfield et al. reported [17] four patient groups were examined and treated with placebo and OCA 10 mg/d, 25 mg/d, and 50 mg/d. Nevens et al. [16] investigated three groups of patients treated with placebo and OCA 5 mg/d titrated 10 mg/d and 10 mg/d, while Kowdley et al. [14] examined three groups treated with placebo and OCA 10 mg/d and 50 mg/d. On the comprehension of weighing the efficacy and safety, the 10 mg/d dose of OCA proved to be the best treatment option for PBC patients. Thus, we used only data on the 10 mg/d dose of OCA for this meta-analysis.

**Figure 2:**
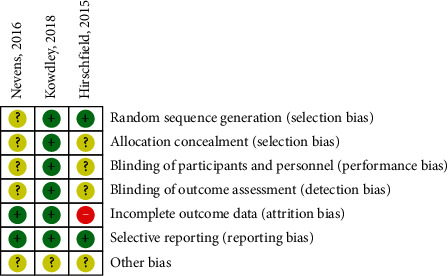
Risk of bias of the included studies evaluated according to each quality item. +, low risk; −, high risk; ?, unclear risk.

**Figure 3:**
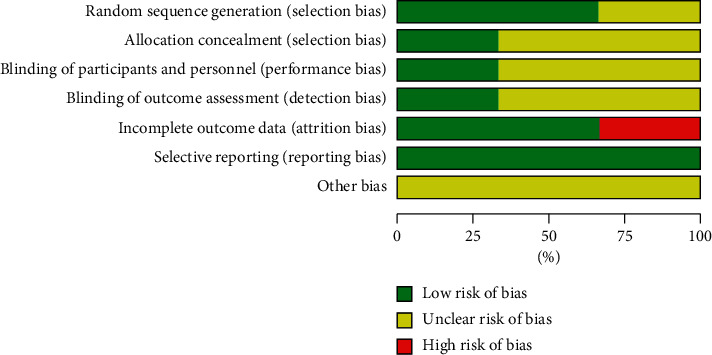
Risk of bias graph in the included studies: authors' judgments for each risk of bias item presented as percentages.

We summarized the risk of bias of the three included trials in Figures 2 and 3.

This meta-analysis involved 442 patients: 265 patients were randomized to the OCA 10 mg/d group and placebo group. The baseline characteristics of the three trials are provided in Table 1. The mean age was about 55 years old, and the mean follow-up interval was 3 or 12 months.

### 3.2. Effect of OCA on Patients with PBC

**Figure 4:**
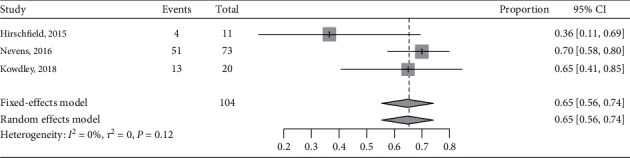
The pooled overall complete response rate in patients with PBC treated with OCA 10 mg/d monotherapy or added to UDCA according to the Paris I criteria.

**Figure 5:**
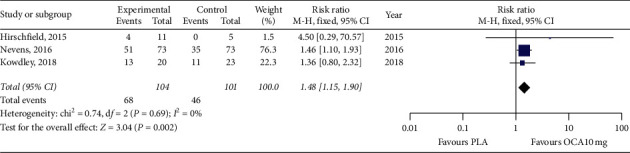
Effect on the overall complete response in patients with PBC treated with OCA versus placebo.

There were 205 patients with PBC evaluated according to the Paris I criteria in the included studies. Among 104 patients treated with OCA 10 mg/d, 68 patients met the Paris I criteria, and a pooled response rate was 65% (95% CI, 56%–74%) (Figure 4). There were significant differences between groups (RR, 1.48; 95% CI, 1.15–1. 90; Figure 5) without heterogeneity (*P*=0.769, *I*^2^ = 0%). OCA's effects on patients with PBC after omitting Hirschfield et al.'s trial due to the incomplete outcomes data and the results were similar (Supplementary Figure S1).

### 3.3. Effect of OCA on Lipids in Patients with PBC

**Figure 6:**
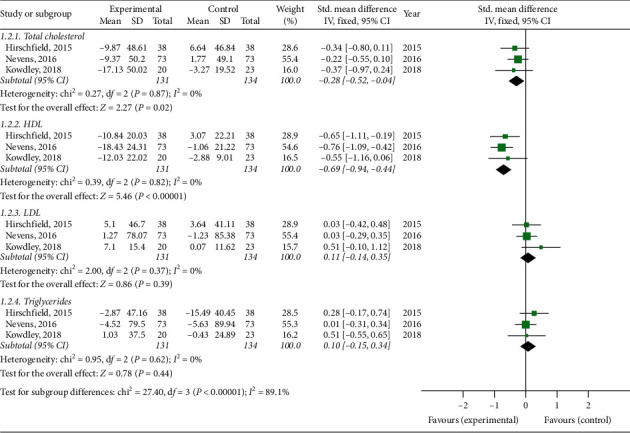
Effects on the lipids level in patients with PBC treated with OCA versus placebo.

Three trials reported the change in total cholesterol, HDL, LDL, and the triglycerides level from baseline to endpoint. In patients with PBC, OCA significantly decreased total cholesterol (*P*=0.02; Figure 6) with no heterogeneity (*P*=0.87, *I*^2^ = 0%) and HDL levels (*P* < 0.05; Figure 6) with no heterogeneity (*P*=0.82, *I*^2^ = 0%). OCA cannot affect the levels of LDL (*P*=0.39; Figure 6) with no heterogeneity (*P*=0.37, *I*^2^ = 0%) and triglycerides (*P*=0.44; Figure 6) without heterogeneity (*P*=0.62, *I*^2^ = 0%) in patients with PBC.

## 4. Discussion

To our knowledge, the article is the first attempt to make a meta-analysis to pool the response rate of OCA treatment based on commonly referenced biochemical response criteria in patients with PBC. The biochemical markers presented as a surrogate for the hard endpoints of mortality [18], which is hard to complete due to PBC's slow nature course. The referenced biochemical treatment response criteria included the vital biochemical markers of PBC. It could be better to evaluate the efficacy of the treatment and predict the prognosis of the disease [18, 19]. Among them, Paris I criteria is one of the common criteria used in clinical trials of PBC treatment [13]. The result showed that the overall complete response rate of OCA 10 mg once daily monotherapy or added to UDCA was 65% in PBC patients, which was superior to the placebo. The meta-analysis reported by Li et al. [20] evaluated OCA's effectiveness by pooling the primary endpoint, which was not one of the commonly referenced biochemical response criteria. The result might be inconvenient for physicians to compare the efficacy between the drugs in clinical practice.

Besides the biochemical response criteria, some clinical characteristics and autoantibodies may be associated with disease prognosis. The clinical characteristics at diagnosis, such as younger age at onset, fatigue, and/or pruritus at diagnosis, are essential for the poor disease prognosis and less response to UDCA treatment [21]. The mean age at diagnosis in patients with PBC was younger, and the 62% of PBC patients who had an inadequate response to UDCA in the study of Nevens et al. had pruritus, which was higher than the percentage of general PBC patients with pruritus (about 33.3%) [7]. These results were consistent with the study of Quarneti et al. [21]. Recent studies have proven that antinuclear antibodies (anti-gp210/antinuclear Rim antibody) positivity has a worse prognosis and less response to UDCA therapy [22–24]. The researchers of the included studies only used autoantibody as one of the diagnostic criteria. Furthermore, we could not get the autoantibody profile data from the included studies of the meta-analysis.

Most patients with PBC are concomitant with hypercholesterolemia, mainly due to raised lipoprotein X [25]. Although there is still a controversial relationship between cardiovascular disease and hypercholesterolemia in patients with PBC [26, 27], hypercholesterolemia is still one of the risk factors for cardiovascular disease [28]. Thus, the change in lipid profiles in patients with PBC needs to be noted. Recent clinical trials showed that OCA could affect lipid profiles of the average population [29] and might have a beneficial impact on metabolic syndrome [30, 31]. However, the effect on lipid profiles of OCA-treated patients with PBC is currently uncertain. Previous studies reported inconsistent results [14, 16, 17]. The meta-analysis results showed that serum total cholesterol and HDL levels decreased in OCA-treated patients with PBC compared to placebo. In contrast, the LDL and triglycerides levels were not significantly different.

OCA is a semisynthetic hydrophobic bile acid analog highly selective for FXR [22] and by FXR activation promotes metabolic regulation [32]. The decrease of the HDL level might be caused by the negative expression of the APOA1 gene modulated by FXR agonists in the study of human apolipoprotein A-1 (APOA1) transgenic mice [33, 34]. Lipid's abnormalities in patients with PBC are complex, related to the stage of liver dysfunction [25]. Many factors could impact the lipid metabolic regulation, so the OCA's effect on lipids in patients with PBC needs further clinical trials.

This study has a few limitations. First, there were limited included studies, and the sample size was limited, resulting in a restricted pooled population in the analysis. It was related to quite a rare prevalence of PBC, but meta-analyses could overcome the shortcoming by pooling more studies. Furthermore, no heterogeneity in the meta-analysis demonstrated that the included studies are statistically similar. Therefore, the pooled results are convincing. Second, biochemical treatment response criteria were not the primary outcomes in all the included studies. The incomplete outcomes data could lead to attrition bias. However, we got a similar result after omitting the trial of incomplete outcomes data. Third, longtime data on clinical outcomes were still absent. Hence, further clinical trials are needed to confirm the results.

## 5. Conclusions

There is an urgent need for developing new treatment options for patients with PBC who had an inadequate UDCA response. This meta-analysis demonstrated that OCA was a promising additional treatment for PBC patients and might reduce serum cholesterol levels. The longer follow-up studies are needed to give more evidence.

## Figures and Tables

**Table 1:** Baseline characteristics of the included studies.

The first author, year	Mean age (year) Mean ± SD	Age at diagnosis (year) Mean ± SD	Number of female (%)	Number of pruritus (%)	OCA dose (mg/day)	Number of patients (OCA 10 mg/d)	Number of controls	Duration of treatment (months)	Publication type
Hirschfield, 2015	56 ± 9	NA	74 (97.4)	NA	10	38	38	3	Published
Nevens, 2016	56 ± 11	47 ± 10	131 (89.7)	91 (62)	10	73	73	12	Published
Kowdley, 2018	54 ± 11	NA	34 (79.1)	NA	10	20	23	3	Published

*Note*. NA, not available.

## Data Availability

The data in the Supplementary Materials files were used to support this study.

## Abbreviations

PBC: Primary biliary cholangitis
UDCA: Ursodeoxycholic acid
OCA: Obeticholic acid
ALP: Alkaline phosphatase
HDL: High-density lipoprotein cholesterol
LDL: Low-density lipoprotein cholesterol
RCT: Randomized controlled trial
FXR: Farnesoid X receptor
JUN: June
FGF-19: Fibroblast growth factor-19
FGF-15: Fibroblast growth factor-15
6-ECDCA: 6-Alpha-ethyl-chenodeoxycholic acid
ULN: Upper limit of the normal range.

## Supplementary Materials

Supplementary Materials Supplementary 1. Electronic search strategy for PubMed database. Supplementary Figure S1. (a) Effect on the biochemical response in patients with PBC treated with OCA versus placebo after omitting the incomplete outcome data. (b) The pooled biochemical response rate in patients with PBC treated with OCA omitting the incomplete outcome data.
