# Supplementary material for: Response Rate and Impact on Lipid Profiles of Obeticholic Acid Treatment for Patients with Primary Biliary Cholangitis: A Meta-Analysis
Source: Can J Gastroenterol Hepatol. 2021 Jan 15;2021:8829510. doi: 10.1155/2021/8829510 (PMC7822683; doi:10.1155/2021/8829510)
Supplement: Supplementary Materials — Supplementary 1. Electronic search strategy for PubMed database. Supplementary Figure S1. (a) Effect on the biochemical response in patients with PBC treated with OCA versus placebo after omitting the incomplete outcome data. (b) The pooled biochemical response rate in patients with PBC treated with OCA omitting the incomplete outcome data. [file 8829510.f1.zip › 8829510.f1/supplement-1[1].docx]

Electronic search strategy for Pubmed database.

#1. Primary biliary cirrhosis

#2. Primary biliary cholangitis

#3. PBC

#4. Obeticholic acid

#5. OCA

#6. farnesoid X receptor

#7.FXR

#8. Fibroblast growth factor-19

#9. FGF-19

#10. Fibroblast growth factor-15

#11. FGF-15

#12. INT-747

#13. 6alpha-Ethyl-chenodeoxycholic acid

#14. 6-ECDCA

#15.#1 or #2 or #3

#16.#4 or #5 or #6 or #7 or #8 or #9 or #10 or #11 or #12 or #13 or #14

#17.#15 and #16
